# Gene amplification during differentiation of mammalian neural stem cells *in vitro* and *in vivo*

**DOI:** 10.18632/oncotarget.3248

**Published:** 2015-02-19

**Authors:** Ulrike Fischer, Christina Backes, Abdulrahman Raslan, Andreas Keller, Carola Meier, Eckart Meese

**Affiliations:** ^1^ Department of Human Genetics, Saarland University, 66421 Homburg/Saar, Germany; ^2^ Department of Anatomy and Cell Biology, Saarland University, 66421 Homburg/Saar, Germany; ^3^ Clinical Bioinformatics, Saarland University, 66123 Saarbrücken, Germany

**Keywords:** neurosphere, genomic stability, in vivo, array-CGH

## Abstract

In development of amphibians and flies, gene amplification is one of mechanisms to increase gene expression. In mammalian cells, gene amplification seems to be restricted to tumorigenesis and acquiring of drug-resistance in cancer cells. Here, we report a complex gene amplification pattern in mouse neural progenitor cells during differentiation with approximately 10% of the genome involved. Half of the amplified mouse chromosome regions overlap with amplified regions previously reported in human neural progenitor cells, indicating conserved mechanisms during differentiation. Using fluorescence *in situ* hybridization, we verified the amplification in single cells of primary mouse mesencephalon E14 (embryonic stage) neurosphere cells during differentiation. *In vivo* we confirmed gene amplifications of the TRP53 gene in cryosections from mouse embryos at stage E11.5. Gene amplification is not only a cancer-related mechanism but is also conserved in evolution, occurring during differentiation of mammalian neural stem cells

## INTRODUCTION

DNA sequence amplification is a phenomenon that occurs predictably at defined stages during normal development in *Xenopus*, *Drosophila*, *Sciara* and *Tetrahymena* [1–4]. A cell's strategy of amplifying genes represents a means of satisfying a heavy demand for stage-specific proteins [1]. These amplifications affect specific DNA regions and appear during narrow windows of development [4]. In mammals, gene amplification appears to be absent in normal cells but commonly occurs in cancer cells. However, we recently published first evidence for gene amplifications during differentiation of human neural progenitor cells [5].

Recent publications on haploid embryonic stem cells reported an intact genome without amplifications and losses. However, comparative genomic hybridization (CGH) data in these studies showed genomic imbalances that were not further investigated due to the selected threshold for amplification detection [6]. Likewise genomic imbalances reported for stem cells and/or induced pluripotent stem cells have always been interpreted based on threshold settings that were consistent with the hypothesis of an intact genome. In addition these imbalances were found between tissue samples including brain, testis, liver and blood samples [7]. These tissues were known to contain stem cells and differentiating cells of varying stage. To explain the genomic imbalances authors frequently blame preparation conditions and the influence of early and late replication timing. As of now, there is, however, no final evidence about the origin of the imbalances that are observed throughout many studies.

Our results on human neural progenitor cells are indicative of amplification as physiological process during stages of differentiation [5]. To follow up on this finding, we set out to investigate the hypothesis that gene amplifications occur as a developmental process in different species. Interestingly double minutes (DMs) as cytogenetic manifestations of gene amplification were found in 1% of serum free mouse embryo (SFME) cells and an increased frequency of DMs was found in cells grown in medium containing fetal calf serum (FCS) [8]. SFME cells were a neural stem cell line consisting of neural progenitor cells that are capable of differentiating into astrocytes when grown with growth factor TGF-ß or fetal calf serum (FCS). SFME cells were routinely cultivated on fibronectin coated culture ware. Many studies however have shown, that cell surface interactions of neural stem cells to extracellular matrix proteins (e.g. fibronectin, laminin) were capable of inducing cell differentiation processes suggesting synergic effects of adhesion and growth factor signals [9]. Sphere growth was reported for SFME cells as unattached multicellular aggregates in the absence of fibronectin [10]. Here we analyzed mouse neural progenitor cells during differentiation using SFME sphere cells and primary mesencephalon E14 neurosphere cells. Since both, our previous human and the present mouse analyses, are performed with cells under *in vitro* differentiation conditions, we also investigate amplifications on mouse embryo tissue sections to provide *in vivo* evidence for gene amplification as a physiological process.

## RESULTS

### Amplification analysis in SFME cells

**Figure 1:**
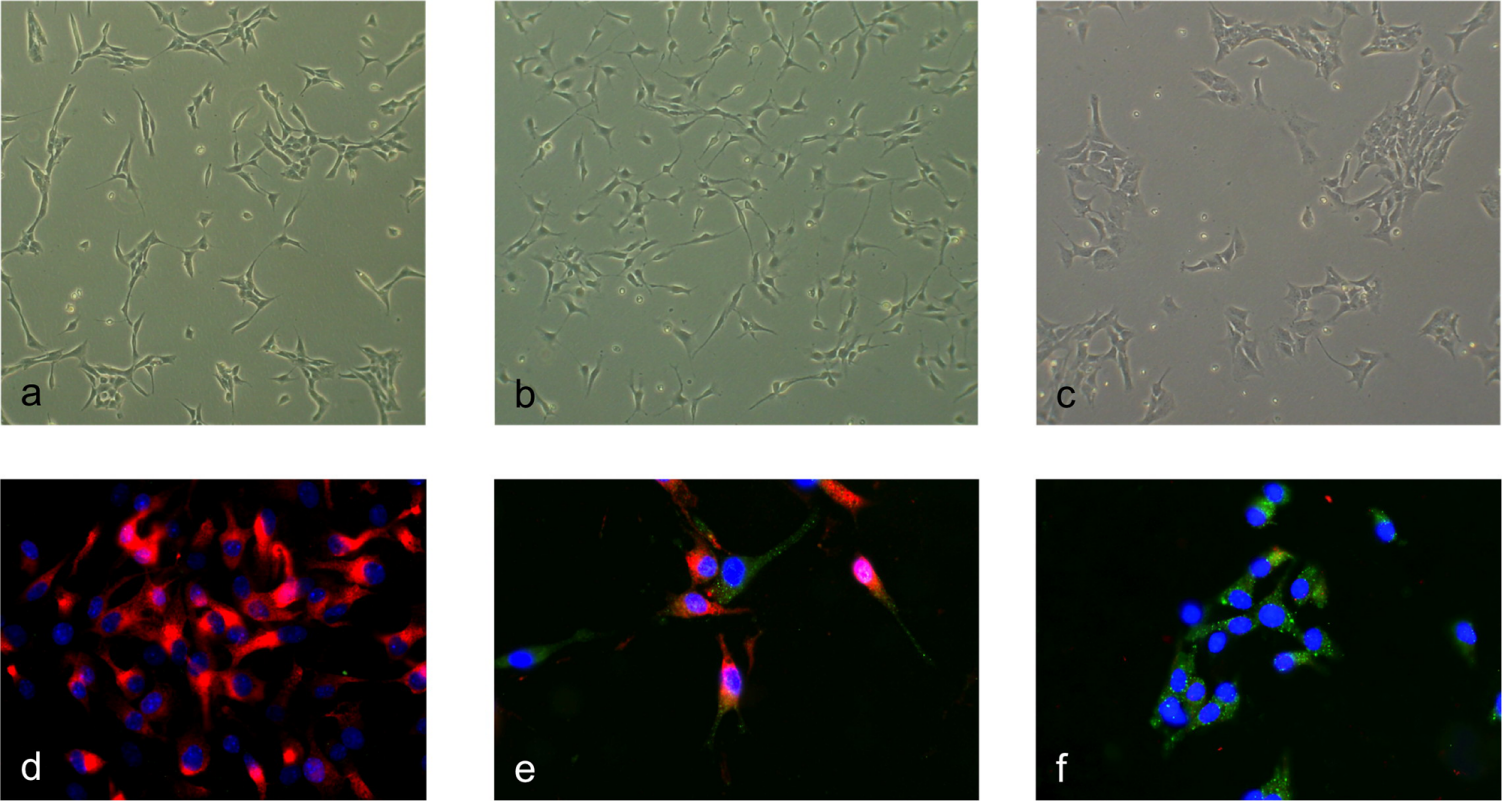
Morphology and marker expression changes upon differentiation induction Undifferentiated SFME cells revealed a fibroblast-like morphology. **(a)** SFME cells differentiation-induced for 24 h by TGF-ß revealed filigree appendages. **(b)** SFME cells differentiation-induced for 12 h by FCS revealed a cobblestone-like morphology. **(c)** Immunofluorescence analysis was done with the neural stem cell marker nestin and glial marker GFAP. Undifferentiated SFME cells solely expressed nestin (red fluorescence). **(d)** TGF-ß differentiation induced SFME cells either expressed both nestin and GFAP, or GFAP only (green fluorescence). **(e)** FCS differentiation induced SFME cells expressed only GFAP. **(f)** Nuclei were counterstained with DAPI.

To identify early differentiation-associated amplifications we performed array-CGH analysis on SFME cells that were induced to differentiate using different conditions. Former studies showed an increased glial differentiation specific *GFAP* mRNA expression 24 h after TGF-ß addition and 8–16 h after FCS addition. Based on these observations we choose to analyze undifferentiated SFME cells grown as spheres, SFME cells grown for 12 h with 10% FCS, and SFME cells grown for 24 h with TGF-ß. As shown in Figure 1a–1c we found clear morphology changes between the treatments. The SFME cells were also analyzed by immune fluorescence (Figure 1d–1f). Undifferentiated SFME cells expressed the neural stem cell marker nestin. Out of the SFME cells that were grown 24 h with TGF-ß, 30% of cells did not show nestin expression but GFAP expression, 50% of cells showed simultaneous nestin and GFAP expression and 10% of cells showed only nestin expression. All SFME cells that were grown for 12 h with 10% FCS showed GFAP expression but no nestin expression.

The array-CGH experiments were done by a two-color approach and all analyzed samples were compared to mouse genomic DNA from Clontech. We established the following data analysis pipeline to determine amplifications: Following the array-CGH analysis, signal intensities were extracted from scanned images of each array using Roche NimbleGen NimbleScan v2.6 software. Intensity values for Cy3 and Cy5 were spatially corrected, normalized using Qspline normalization, and the log_2_ of the ratios from Cy3/Cy5 intensity values were calculated. To reduce size and noise of the data we applied a 10 x window-averaging step. To detect amplifications we used the dynamic segMNT algorithm that identifies segments by minimizing the squared error relative to the segment mean. To detect representative alterations and to minimize the identification of random alterations, we extracted segments with segment means above a 0.1 threshold and a size greater than 500 kb. Since we did not compare the SFME cells to the original parental strain (BALB/c) but to normal mouse cells DNA, we expected several CNVs (copy number variations) between the individual mouse samples. We therefore excluded chromosomal regions representing CNVs from our results across all investigated samples that were included in a list of CNVs published in 2010 [11]. For the identification of differentiation-associated amplifications we further excluded chromosomal regions that revealed a copy number gain with a comparable log_2_ ratio value in all samples.

**Figure 2:**
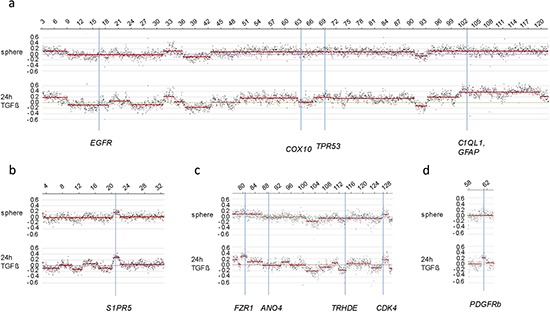
Relative copy number profiles on mouse chromosomes 11, 10, 9 and 18 CGH analysis of SFME cells grown as sphere or differentiation induced with TGF-β revealed multiple amplified and under-replicated regions on mouse chromosome 11 **(a)**, mouse chromosome 9 **(b)**, mouse chromosome 10 **(c)** and mouse chromosome 18 **(d)**. Relative copy number is plotted at 40 kb resolution using a log_2_ scale. Localization of BAC clones used for FISH analysis were indicated including known genes: *C1QL1, GFAP* (RP23–235I12), *TRP53* (RP23–150N14), *EGFR* (RP23–51E21), and *COX10* (RP23–40P10) for chromosome 11; *S1PR5* (RP23–4A11) for chromosome 9; *FZR1* (RP23–421E11), *CDK4* (RP23–432F11), *ANO4* (RP23–279E23), and *TRHDE* (RP23–361I2) for chromosome 10; *PDGFRb* (RP23–143A24) for chromosome 18.

In total we detected 3 amplified regions in undifferentiated SFME sphere cells and 89 amplified chromosomal regions in 24 h-TGF-β-differentiation induced SFME cells. Amplifications were detected on all autosomal chromosomes with no specific clustering on a specific chromosome. In 12 h-FCS-differentiation induced SFME cells we detected only 13 amplified regions. Interestingly 2 out of 13 chromosome regions were solely detectable in FCS-differentiation induced cells and 11 of 13 chromosome regions were detectable under both differentiation induction conditions. The size of the amplified chromosome region varied between 250 kb–22 Mb. Amplified regions are summarized in Table 1. Representative array-CGH plots for mouse chromosomes 11, 9, 10 and 18 are shown in Figure 2a, 2b, 2c and 2d.

**Table 1:** Overview on amplified mouse chromosome regions

SSP1					24 h TGFß					12 h FCS					human		
chr	Start	end	log_2_	size	chr	start	end	log_2_	size	chr	start	end	log_2_	size	chr	start	end
					chr1	35939999	36899999	0.22169	960						chr2	96527664	97828803
										chr1	65659999	66219999	0.15488	560	chr2	209345284	209995048
					chr1	71939999	75659999	0.14225	3720						**chr2**	**216237767**	**220318447**
					chr1	84859999	95819999	0.13328	10960						chr2	230522918	242393106
					chr1	120139999	123499999	0.14003	3360						chr2	118238123	122295007
					chr1	153739999	157659999	0.17938	3920						chr1	178390600	182855809
					chr1	180179999	185379999	0.11946	5200						chr1*	242936091	245192366
					chr2	3019999	6179999	0.104	3160						chr10	11763772	15489267
					chr2	24779999	33699999	0.22398	8920						chr9*	**129144754**	**140509852**
					chr2	69219999	71539999	0.11333	2320						chr2	169636341	172911598
					chr2	90019999	94379999	0.15133	4360						chr11	43165295	48161984
					chr2	101219999	102579999	0.22708	1360						chr11	35290418	36788724
					chr2	116539999	120579999	0.13508	4040						**chr15**	**35850202**	**40862514**
					chr2	150339999	158859999	0.15123	8520						**chr20**	**24871670**	**37312457**
					chr2	162699999	168379999	0.19985	5680						chr20	41442652	49543434
					chr2	178859999	181732904	0.10906	2873						**chr20**	**58855603**	**62378035**
					chr3	27339999	28339999	0.13389	1000						chr3	172483565	173561421
					chr3	37499999	38659999	0.20343	1160						chr4	124492293	126216185
					chr3	50859999	52339999	0.10696	1480						chr4	139957187	141410919
					chr3	87699999	88539999	0.25357	840						**chr1**	**154134173**	**154997209**
					chr3	88739999	90539999	0.16515	1800						**chr1**	**151579369**	**153953612**
chr3	96299999	96899999	0.13071	600	chr3	94099999	98379999	0.11506	4280						chr1	119886581	150154226
					chr3	106859999	109139999	0.11082	2280						chr1	108313206	110995784
					chr4	39899999	41739999	0.11301	1840						chr9	32191776	34658184
					chr4	42779999	47299999	0.12366	4520						chr9	34691224	100844257
					chr4	114019999	120779999	0.13465	6760						**chr1**	**40488788**	**48298600**
					chr4	122459999	144979999	0.17507	22520						**chr1**	**12018456**	**40343625**
					chr4	147219999	155615021	0.13332	8395						**chr1**	**884178**	**12014935**
					chr5	23779999	26339999	0.15304	2560						**chr7**	**150232064**	**152310085**
					chr5	29739999	37619999	0.11759	7880						chr2	26247649	28877966
					chr5	64259999	67659999	0.10447	3400						chr4	37210722	41641811
					chr5	110459999	119019999	0.17299	8560						chr12*	**115187887**	**119827164**
					chr5	120459999	126139999	0.22594	5680						chr12*	**120534293**	**124505940**
					chr5	128459999	134419999	0.10258	5960						chr7	66668494	71579817
					chr5	134459999	138779999	0.25283	4320						chr7	99807657	101978853
					chr5	139219999	141499999	0.24122	2280						**chr7**	**477588**	**3074849**
					chr5	142499999	145899999	0.1965	3400						chr7	97436244	98843217
chr6	47379999	48459999	0.11889	1080											chr7	147992524	149174079
					chr6	112059999	116019999	0.18538	3960						**chr3**	**8337660**	**12858927**
					chr7	50699999	54859999	0.27538	4160						chr19*	53491829	56560862
					chr7	70699999	74739999	0.11528	4040						chr15*	97665038	100083393
					chr7	103379999	110579999	0.12746	7200						chr11	71304680	78810202
					chr8	9699999	13899999	0.12003	4200						chr13	107240678	113373977
					chr8	72059999	75499999	0.16997	3440						**chr19**	**17831477**	**19635937**
					chr8	86139999	88059999	0.20128	1920						**chr19**	**12618314**	**14480321**
					chr8	96419999	98699999	0.16163	2280						**chr16**	**54867237**	**57639190**
					chr8	106459999	114739999	0.11054	8280						chr16*	**66219587**	**75978057**
					chr8	117859999	125699999	0.14206	7840						**chr16**	**77786623**	**88207643**
					chr8	125739999	126379999	0.35041	640						chr16*	89728131	90110030
					chr9	20299999	21979999	0.2457	1680						**chr19**	**9780537**	**11550880**
					chr9	36339999	37379999	0.12983	1040						chr11	124097804	125060128
					chr9	40019999	46259999	0.13034	6240						chr11	115944719	123101315
					chr9	56299999	62059999	0.13931	5760						**chr15**	**67158620**	**73815792**
					chr9	62099999	62619999	0.32652	520						chr15	66393109	67077238
					chr10	74339999	78019999	0.15637	3680	chr10	76939999	78219999	0.10128	1280	**chr21**	**43963339**	**46909340**
					chr10	79019999	81099999	0.27325	2080	chr10	79019999	80819999	0.10647	1800	**chr19**	**312979**	**2448327**
					chr10	126179999	128499999	0.15741	2320						**chr12**	**54251773**	**56822806**
					chr11	3019999	8939999	0.15324	5920						chr22*	**29167187**	**32022116**
					chr11	31499999	33939999	0.19246	2440	chr11	33379999	33939999	0.2177	560	chr5*	172963280	173711408
					chr11	49419999	63059999	0.13883	13640						chr5*	150381644	154347235
					chr11	66659999	70979999	0.15978	4320						**chr17**	**6499209**	**10751699**
					chr11	71139999	90459999	0.1263	19320						chr17*	**25556525**	**34455508**
					chr11	93339999	100859999	0.1662	7520						chr17*	**36351926**	**40589548**
					chr11	100899999	119819999	0.34969	18920	chr11	101459999	104179999	0.25896	2720	**chr17**	**38734877**	**41444122**
										chr11	104219999	105099999	0.15425	880	**chr17**	**41501998**	**57973933**
										chr11	105739999	108219999	0.24245	2480	chr17*	**61720138**	**63614987**
										chr11	108659999	109899999	0.2712	1240	chr17*	60746591	61126062
										chr11	109939999	111219999	0.16527	1280	chr17	64469027	65955533
										chr11	111979999	116499999	0.27403	4520	**chr17**	**66882022**	**72015100**
										chr11	117059999	119819999	0.26674	2760	**chr17**	**72789014**	**76642156**
					chr11	119859999	121841731	0.19144	1982						**chr17**	**76694524**	**78652610**
					chr12	3019999	5299999	0.15378	2280						**chr2**	**23550202**	**26214214**
					chr12	55779999	56659999	0.29179	880						chr14	34068138	35042196
					chr12	69859999	89899999	0.10143	20040						**chr14**	**48746376**	**77659229**
					chr13	51379999	55939999	0.14561	4560						chr5*	174293798	176972836
chr13	68179999	68699999	0.15442	520											chr5*	7971780	7978769
					chr13	115099999	115899999	0.28021	800						chr5	52111972	52988491
					chr14	24579999	26659999	0.19976	2080						**chr10**	**78790148**	**80925449**
					chr15	36139999	39019999	0.14402	2880						chr8	101294130	104569815
					chr15	57139999	59659999	0.11708	2520						**chr8**	**123420009**	**126725912**
					chr15	77419999	86459999	0.19653	9040						chr22	34957607	46133888
					chr15	88379999	89419999	0.17115	1040						**chr22**	**48415099**	**49566770**
					chr15	97299999	103486909	0.14593	6187						**chr12**	**46049365**	**53335723**
					chr16	29379999	35459999	0.12251	6080						chr3*	124300383	126805338
										chr16	47819999	48699999	0.10891	880	chr3	110097591	110989426
					chr17	3019999	3579999	0.19459	560						chr6	155094775	155696551
					chr17	5259999	6219999	0.15669	960						chr6	157469658	158830969
					chr17	6939999	15099999	0.10731	8160						chr6	160023022	167303093
					chr17	23579999	27339999	0.18833	3760						**chr16**	**166593**	**3140258**
					chr17	27579999	30579999	0.21704	3000						chr6	34122007	38556691
					chr17	31139999	35499999	0.1682	4360						chr6	31429388	33485622
					chr17	35619999	36459999	0.24316	840						chr6	30335245	31251928
					chr17	45499999	47979999	0.2613	2480						**chr6**	**41705421**	**44564163**
					chr17	56059999	57499999	0.20329	1440						**chr19**	**4180082**	**6808452**
					chr17	73139999	76099999	0.11553	2960						chr2	30157660	33853420
					chr17	80099999	81459999	0.18623	1360						chr2	38137499	39856920
					chr18	33979999	36939999	0.16522	2960						**chr5**	**137252984**	**140048188**
					chr18	37979999	39259999	0.16886	1280						**chr5**	**140849662**	**142237134**
					chr18	60779999	61659999	0.21367	880	chr18	60779999	61659999	0.10276	880	**chr5**	**148986334**	**149989065**
					chr19	3019999	9219999	0.17944	6200						**chr11**	**61861339**	**68466298**
					chr19	43659999	47739999	0.17313	4080						**chr10**	**101249361**	**105802462**

Start and end point of mouse amplified chromosome regions were according to NCBI37/mm9 and converted human sequences were according to NCBI36/HG18. Converted human sequences matching amplified human chromosome regions were displayed in bold. Human sequences marked by an asterisk were only partially converted. Sizes were displayed in kb.

**Figure 3:**
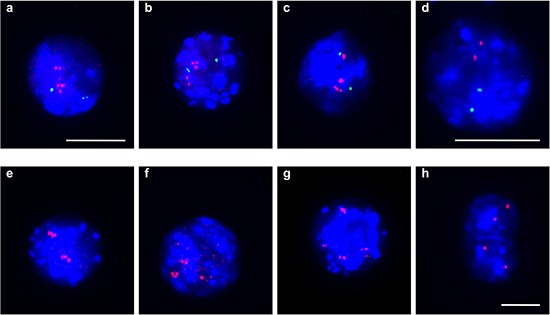
Gene amplifications on mouse chromosomes 11 and 9 on SFME cells Interphase-FISH was used to confirm gene amplifications for *C1QL1* on interphase nuclei from 24 h-TGF-β–differentiation induced SFME cells **(a–c)**. *C1QL1* specific BAC-probe (RP23–235I12) was labeled in pink and control BAC probe (RP23–51E21) (containing *EGFR*) from the same chromosome was labeled in green. Representative interphase nuclei without *C1QL1* amplification from not-differentiated cells revealed comparable fluorescence signals for *C1QL1* and *EGFR* control probe **(d)** Interphase-FISH was used to confirm gene amplifications for *S1PR5* on interphase nuclei from 24 h TGF-β*-*differentiation induced cells **(e–g)**. *S1PR5* specific BAC-probe (RP23–4A11) was labeled in pink. Representative interphase nuclei from undifferentiated cells revealed single copy fluorescence signals for gene *S1PR5* **(h)** Nuclei were counterstained with DAPI. Size calibration bar = 5 μm.

As an independent method of verifying amplification in single cells, we used fluorescence *in situ* hybridization (FISH). Representative BAC probes used for FISH analysis were selected for both the amplified and the control region. Corresponding gene names were only used for annotation of the BAC probes. Localization of probes used for FISH experiments are indicated in Figure 2. Two BAC probes were selected for two amplified chromosome regions on two different chromosomes with a log_2_ ratio value greater 0.24 (Table 1). We selected one region solely amplified in TGF-ß differentiation induced cells on chromosome 9 and one region amplified under both differentiation-induction conditions on chromosome 11. Both amplified chromosome regions were confirmed to be amplified using fluorescence *in situ* hybridization on individual cells. Examples of amplification of sequences on mouse chromosome 11q detected in 24 h-TGF-β–differentiation induced cells are illustrated in Figure 3a–3c. Amplifications were seen on interphase nuclei revealing several fluorescence signals for BAC RP23–235I12 (including *C1QL1* gene in pink) and two fluorescence signals for the control probe BAC RP23–51E21 (including *EGFR* gene in green). An example of normal copy number fluorescence signals for BAC RP23–235I12 and the control probe BAC RP23–51E21 is shown in Figure 3d. Examples of sequence amplification on mouse chromosome 9q detected in 24 h-TGF-β–differentiation induced cells are illustrated in Figure 3e–3g. Amplifications were seen on interphase nuclei revealing several fluorescence signals for BAC probe RP23–4A11 (including *S1PR5* gene in pink). Examples of normal copy number fluorescence signals for BAC RP23–4A11 are shown in Figure 3h.

**Figure 4:**
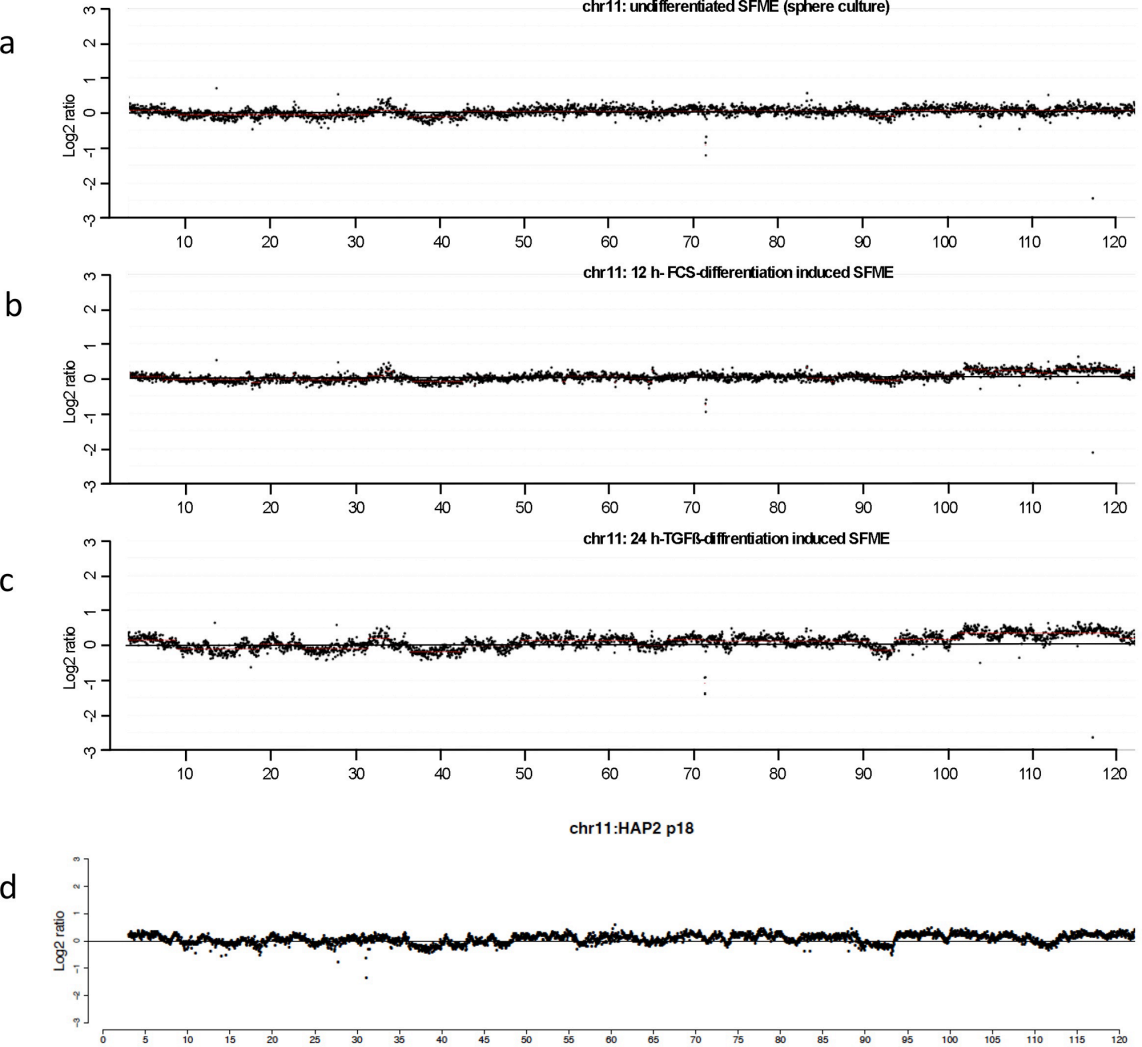
Comparative analysis of the ratio profiles from chromosome 11 indicating similarities and differences of the relative copy number CGH results are displayed as representative chromosome 11 plots at 40 kb resolution using a log_2_ scale. Plots from undifferentiated sphere SFME cells revealed an almost smooth pattern of the ratio profile **(a)**. Plot from 12 h-FCS differentiation induced SFME cells showed a similar smooth pattern of ratio profile with increased ratios for chromosome region 104–120 Mb **(b)**. Plot from 24 h-TGF-ß differentiation induced SFME cells revealed a highly wavy pattern indicating multiple genomic imbalances with increased ratios for chromosome region 104–120 Mb **(c)**. The wavy pattern visible in (c) revealed similarities to the wavy pattern of haploid embryonic stem cell line HAP2 recently published in Nature by Leeb and Wutz 2011 (Supplementary Figure) **(d)**.

Taken together, in differentiation induced SFME we determined a wavy pattern of genomic imbalances including amplifications that were confirmed in individual cells by fluorescence-*in-situ* hybridizations. Comparing our results with previously published results on array-CGH in haploid embryonic stem cells revealed noteworthy similarities. Representative chromosome plots for chromosome 11 from SFME array-CGH were presented in Figure 4a–4c. The pattern of imbalances present in TGF-ß-differentiation induced SFME cells is very similar to the pattern of imbalances at chromosome 11 in haploid embryonic stem cells published by Leeb and Wutz 2011 as presented in Figure 4d. This observation illustrates that far more stem cells use the mechanism of gene amplification during their development than hitherto believed.

Additional chromosome plots for chromosome 11 from array-CGH analysis at various time points and differentiation induction conditions were supplied in a Supplementary Figure S1a–S1d.

### Analysis of amplified regions in mice

Previously, we reported 66 amplified chromosome regions after a 2 day long differentiation induction and 93 amplified chromosome regions after a 5 day differentiation induction in human neural progenitor cells [5]. We ask if and to what extend the human and the mouse amplifications overlap on the chromosomal level. Using UCSC-Genome Browser we converted mouse chromosome regions amplified in SFME sphere cells and FCS and/or TGF-β-differentiation induced SFME cells to the corresponding human chromosomal regions. Several regions failed to convert completely because sequences were split or duplicated. Out of 92 converted mouse sequences, 46 showed an overlap with amplified regions found in human neural progenitor cells after 5 day differentiation induction and 3 regions showed an overlap with amplified regions in human neural progenitor cells after 2 day differentiation induction. A detailed overview on this conversion is given in Table 1.

### Gene amplification analysis on primary neural stem and progenitor cells

**Figure 5:**
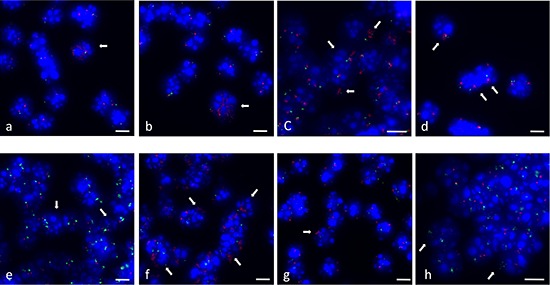
Gene amplifications in differentiation induced neurosphere cells FISH was used to confirm gene amplifications of six gene loci on nuclei from differentiation induced mesencephalon E14 neurosphere cells. *FZR1* (RP23–421E11) (pink) gene amplification was confirmed in 2-day differentiation induced cells and normal copy number of the control probe from the same chromosome *ANO4* (RP23–279E23) (green) **(a, b)**. *FZR1* (pink) amplifications detected in 3-day differentiation induced cells frequently revealed an increase of fluorescence signals at only one of the corresponding loci **(c)**. *CDK4* (RP23- 432F11) (pink) gene amplification in 3-day differentiation induced cells. Hybridization of the control gene (from the same chromosome) *TRHDE* (RP23–361I2) (green) revealed decreased fluorescence signal intensity **(d)**. *TRP53* (RP23–150N14) (green) gene amplification was confirmed in 3-day differentiation induced cells with normal copy number of control gene *COX10* (RP23–40P10) (pink) from the same chromosome **(e)**. *C1QL1* (RP23–235I12) (pink) gene amplification was confirmed in 3-day differentiation induced cells with normal copy number of control gene *COX10* (green) **(f)**. *S1PR5* (RP23–4A11) (pink) gene amplification in 3-day differentiation induced cells with normal copy number of control gene *COX10* (green) (different chromosome) **(g)**. *PDGFRb* (RP23–143A24) (green) gene amplification was confirmed in 3 day-differentiation induced cells with normal copy number of control gene *COX10* (pink) (different chromosome) **(h)**. Nuclei were counterstained with DAPI. Size calibration bar = 5 μm. Arrows point to examples of nuclei with amplifications.

To exclude cell culture artifacts in the SFME neural stem cell line we analyzed gene amplifications in primary mesencephalon E14 neurosphere cells at passage P1 using FISH. For amplification confirmation we selected 6 amplified chromosome regions from four different chromosomes on the basis of log_2_ ratio value in array-CGH experiment. Representative BAC probes used for FISH analysis were selected for both the amplified and the control region. Localization of hybridization probes for amplified loci and control probes from the same chromosome are shown in Figure 2 and further information including the percentage of cells with amplification is summarized in Table 2. Undifferentiated neurosphere cells revealed normal copy number in 100 analyzed nuclei. We induced differentiation in primary mesencephalon E14 neurosphere cells for 2 or 3 days in differentiation medium containing small amounts of FCS as supplied by the manufacturer. Hybridization of *FZR1* against neurosphere cells differentiated for 2 days (Figure 5a, 5b) and for 3 days (Figure 5c) revealed an increased number of fluorescence signals scattered over the whole nucleus in contrast to two fluorescence signals from the control probe *ANO4*. The increased number of fluorescence signals along with the two signals found for the control probe confirmed the amplification of *FZR1*. Gene amplifications of *CDK4* after 3 days of differentiation induction are shown in Figure 5d. Gene amplifications of *TRP53* (Figure 5e), *C1QL1* (Figure 5f), *S1PR5* (Figure 5g) and *PDGFRb* (Figure 5h) found in 3-day differentiation induced neurosphere cells confirmed our array-CGH results.

**Table 2:** Details for chromosome regions selected for FISH-confirmation of amplification

Position (mm9)	Position (HG18)	log_2_ ratio value	BAC	Representative gene name	Percentage of cells with amplification
9	20299999–21979999	0.2457	RP23-4A11	*S1PR5*	*15%*
10	79019999–81099999	0.2733	RP23-421E11	*FZR1*	*10%*
10	126179999–128499999	0.1574	RP23-432F11	*CDK4*	*14%*
11	66659999–70979999	0.1598	RP23-150N14	*TRP53*	*5%*
11	100899999–119819999	0.3497	RP23-235I12	*C1QL1 and GFAP*	*33%*
18	60779999–61659999	0.2137	RP23-143A24	*PDGFRb*	*10%*

### Simultaneous gene amplification and GFAP expression

**Figure 6:**
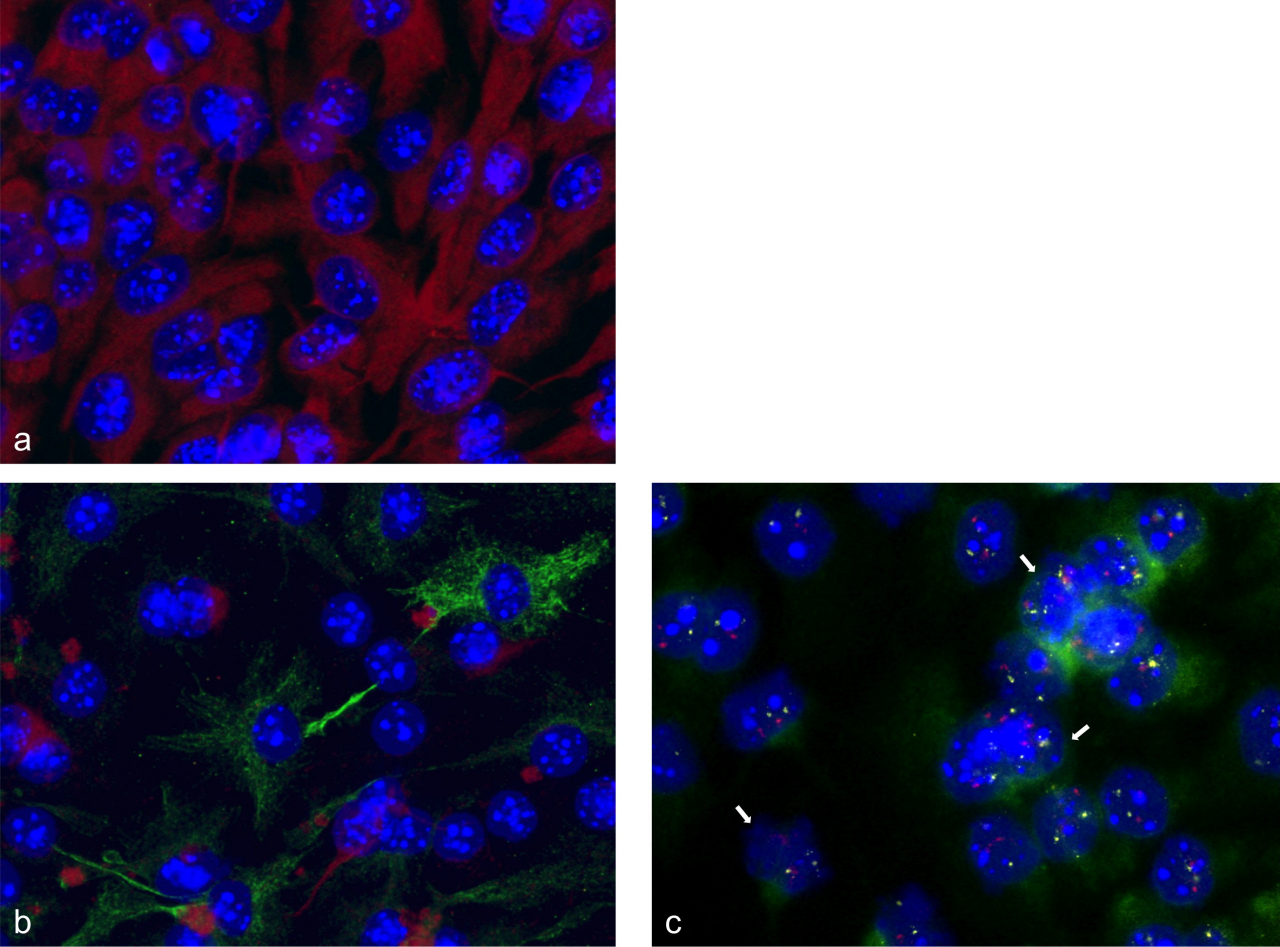
Immune fluorescence and FISH analysis Neurosphere cells express nestin only (red fluorescence) **(a)**; differentiation induced neurosphere cells express GFAP (green fluorescence) and showed a decreased nestin expression (red fluorescence) **(b)**; Simultaneous fluorescence *in situ* hybridization and immune fluorescence analysis showed gene amplifications of *FZR1* (RP23–421E11) (pink fluorescence) and *GFAP/C1QL1* (RP23–235I12) (yellow fluorescence) in mesencephalon E14 neurosphere cells that were differentiation-induced for 2 d. These cells also expressed the GFAP protein (green fluorescence). GFAP expression was present in areas of the cells that developed appendages **(c)**. Nuclei were counterstained with DAPI. Arrows point to examples of nuclei with amplifications.

Immune fluorescence was used to analyze the differentiation of neurosphere cells to glial lineage cells by simultaneous analysis of nestin and GFAP. As shown in Figure 6a neurosphere cells expressed nestin only. As shown in Figure 6b differentiation induced neurosphere cells expressed GFAP and showed a decreased nestin expression. To analyze a possible association between gene amplification and differentiation, we performed simultaneous FISH-analysis and immune fluorescence staining with an antibody against the astrocyte marker protein GFAP. Mesencephalon E14 neurosphere cells that were differentiation-induced for 2 days, showed amplifications for BAC RP23–421E11 containing *FZR1 and* BAC RP23–235I21 containing *C1QL1* and *GFAP.* The differentiation-induced mesencephalon cells showed also GFAP protein expression (Figure 6c).

### Multiple amplification analysis

**Figure 7:**
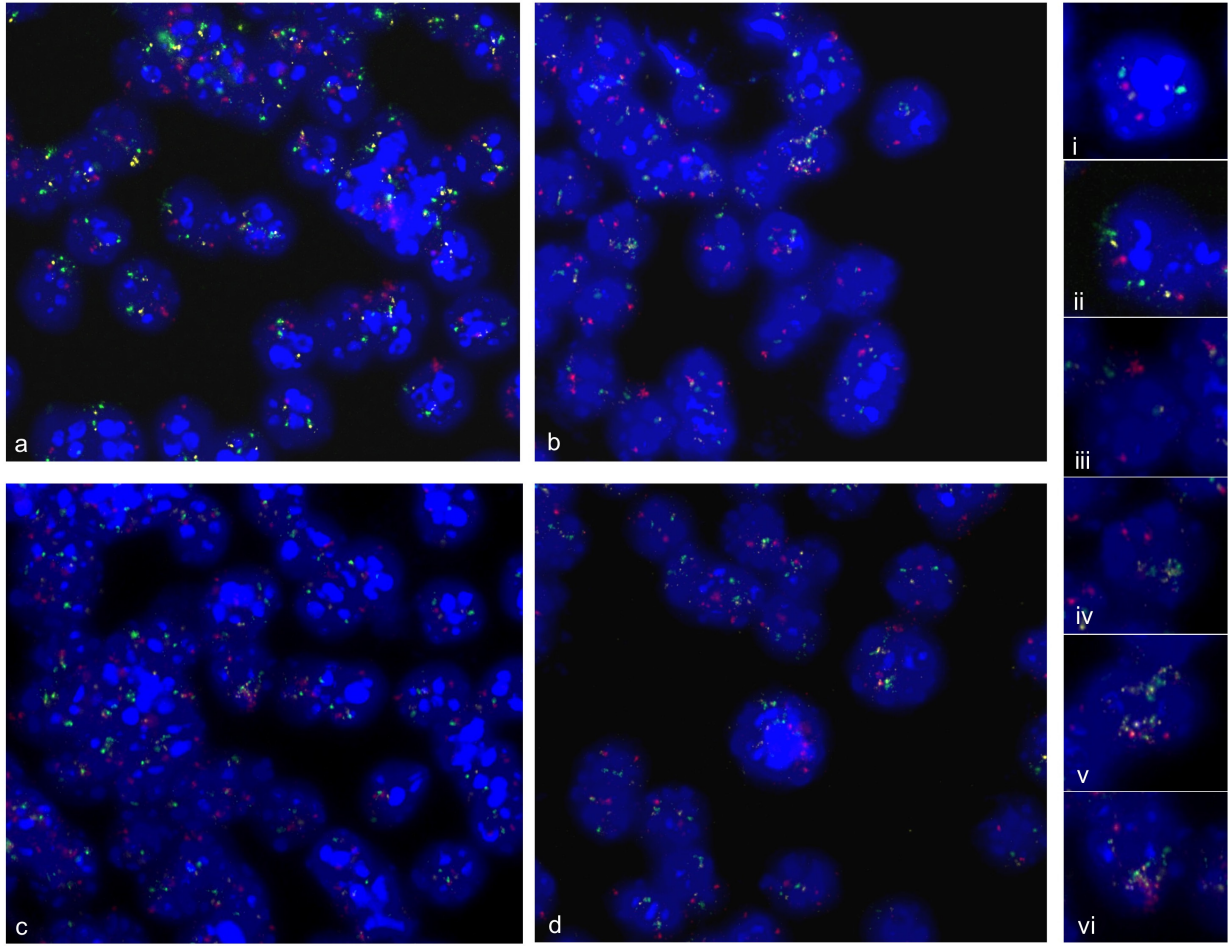
Multiple amplification analysis Simultaneous fluorescence *in situ* hybridization of three genes from amplified chromosome regions on 2 d—differentiation induced neural progenitor cells. BAC probe *C1QL1* (RP23–235I12) was labeled with Alexa-555 in yellow, BAC probe (RP23–150N14) *TRP53* labeled with Alexa-488 in green, and BAC probe (RP23–421E11) *FZR1* labeled with Alexa-594 in pink. An overview on hybridization results is shown in **(a–d)**. Representative examples of fluorescence signal pattern were displayed as enlarged views of nuclei. We found normal copy number of *C1QL1*, *TRP53* and *FZR1* (i), *TRP53* amplification (ii), *FZR1* amplification (iii), *C1QL1* and *TRP53* co-amplification (iv), *TRP53* and *C1QL1* and FZR1 co-amplification (V, Vi). Nuclei were counterstained with DAPI.

We also investigated whether cells revealed multiple amplified chromosome regions per single cell. For co-hybridization experiments we labeled *FZR1* from chromosome 10 with Alexa-594 in pink, *TRP53* from chromosome 11 with Alexa-488 in green, and *C1QL1* from a second amplified region on chromosome 11 with Alexa-555 in yellow. As shown in Figure 7a–7d a very variable pattern of hybridization signals was detectable. We found cells with normal copy number for all investigated probes (i) cells with amplification of either *TRP53* (ii) or *FZR1* (iii) amplification and cells with co-amplification of probes from chromosome 11 (iv) or co-amplification of all investigated probes (v and vi).

### *In vivo* analysis of gene amplification

**Figure 8:**
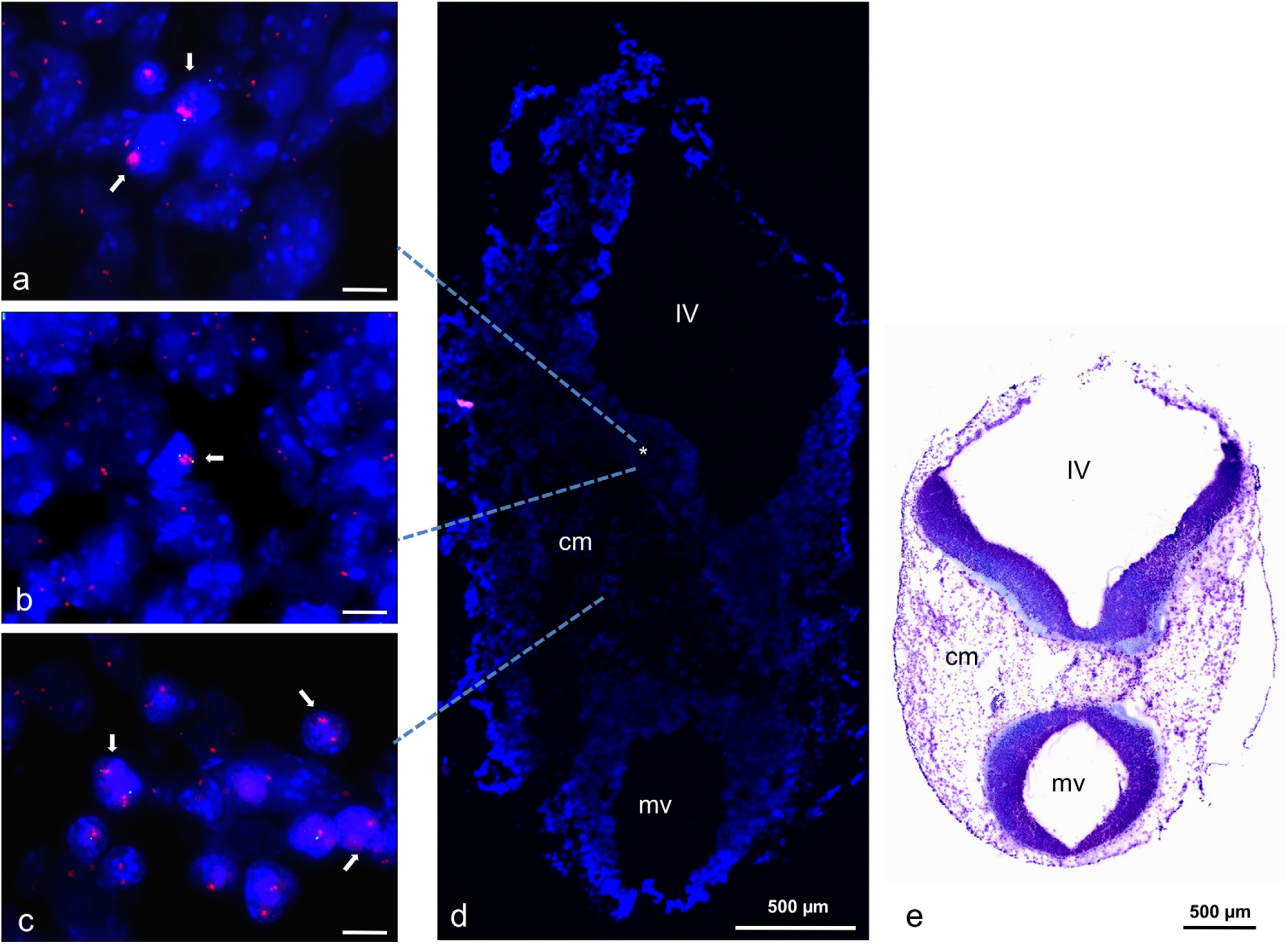
TRP53 amplification in mouse embryo stage E11.5 Amplification identified by FISH on transversal cryosection from mouse head region. High copy number of *TRP53* is indicated by pink fluorescence signals (either enlarged signals and/or multiple signals) and normal copy number of *COX10* by green signals. Both genes (BAC probes) were localized on mouse chromosome 11. Individual cells with amplifications were detected in multiple regions of the metencephalic part of the rhombencephalon (*) **(a, b)** and clustered cells with amplifications were detected in the cephalic mesenchyme (cm) **(c)**. Nuclei were counterstained with DAPI. Size calibration bars in a–c = 5 μm. Arrows point to examples of nuclei with amplifications. Dashed lines point to areas where the pictures were taken from. For orientation, an enlarged DAPI stained overview of the transversal section is shown in **(d)** and a LFB/CV (Luxol Fast Blue und Cresyl Violet) histological stain from an analogical section is shown in **(e)**. IV: fourth ventricle, mv: mesencephalic ventricle, cm: cephalic mesenchyme.

For *in vivo* analysis we selected a chromosome region that was amplified in mouse and human neural progenitor cells during *in vitro* differentiation. We selected *TRP53* for further confirmation as the most prominent gene. Embryonic stage E11.5 was selected because we had preliminary evidence that amplifications occur during stages of brain development. Using FISH analysis we were able to confirm amplification of *TRP53* on a cryosection from a mouse embryo at stage E11.5 (Figure 8a–8c). *TRP53* amplifications were detectable in the developing brain, particularly in cells of the metencephalic part of the rhombencephalon and in cells of the cephalic mesenchyme (Figure 8d, 8e).

## DISCUSSION

Gene amplification during differentiation was recently detected in human neural progenitor cells [5]. Here we report a similar complex amplification pattern in mouse neural progenitor cells during differentiation. Although gene amplifications were mainly an attribute of tumor cells and drug resistant cells in humans, there were many reports of programmed gene amplification during development of amphibians and flies. Interestingly polyploidization in mammalian cells gains more and more attention in respect to its physiological significance and distribution [12]. The reasons for polyploidy were manifold and sometimes achieved by multiple rounds of replication without entering mitosis (endocycles). The occurrence of endocycles is widespread in nature and points to an ancient and practical innovation [13]. In somatic follicle cells of *Drosophila* polyploid cells cease their genome replication and start to endoreplicate four specific genomic regions resulting in gene amplifications [14]. In the present study TGF-β–differentiation induced SFME cells revealed 89 amplified chromosome regions by array CGH analysis. Interestingly, FCS differentiation induced SFME cells show only 13 amplified chromosome regions. These results strongly argue against a simple artifact of DNA preparation or replication timing. In both scenarios one would expect genomic imbalances throughout the genome and not on specific chromosomes. As shown in other studies, a wavy CGH pattern was even found after increased protein digestion, further indicating that the wavy pattern that indicate genomic imbalances was not due to preparation effects [7]. In addition, it is very unlikely that early and late replication effects were responsible for the wavy pattern of amplifications in our experiments since we investigated a very heterogeneous cell population. This effect may apply to rapidly proliferating cells but not to a non-synchronized cell population during differentiation. Although induction of differentiation might have a synchronization effect we did not find an amplification pattern indicative of synchronization. The simultaneous analysis of multiple amplified regions revealed a very heterogeneous pattern of amplifications and co-amplifications that argues against synchronization. In a synchronized cell population one would expect a similar frequency for cells to be involved in amplification of a given gene. We found that the amplification frequency varies between 5% for *TRP53* and 33% for *C1QL1* and *GFAP*. One would also expect that cells with amplifications would have the same regions amplified and/or co-amplified. However we have cells with and without co-amplifications. One would further expect an almost similar intensity of the fluorescence signals for the amplified gene. Here, we have a great variation of fluorescence signal intensities between cells with an amplified gene.

As shown in Supplementary Figure S1 we found amplifications and an increased wavy pattern in SFME cells freshly seeded on fibronectin and cultivated for one day. Although still proliferating the SFME cells cultivated for 4 d revealed a more flattened pattern. This observation suggests an early influence mediated by fibronectin on differentiation-induced amplifications.

Interestingly, we found that 50% of the amplified mouse chromosome regions detected after TGF-β differentiation induction overlap with amplified chromosomal regions detectable in human neural progenitor cells upon differentiation induction for 2 and 5 days. The overlap of the amplified chromosome regions between both cell types points to a basic and conserved mechanism used by cells during differentiation. Out of the large number of genes that were affected by amplification, we would like to draw attention to the following groups of genes. Many amplified genes were involved in replication initiation or endocycling, including *GINS* genes, *POLA2*, *PRIM1*, *CDH1*, *FZR*, and genes from the CST (Cdc13-Stn1-Ten1) complex. Recently, a study reported that human CST complex is involved in replication restart [15]. Amplification of *TP53* in human and mouse neural progenitor cells is consistent with previous observation that p53 expression might prevent neuronal terminal differentiation in neuroblasts [16].

There are several lines of evidence pointing to gene amplification as a mechanism to elevate the abundance of mRNAs and proteins in mammalian cells specifically during differentiation. Gurok et al 2004 investigated the mRNA expression pattern of mouse neurosphere cells differentiated by BDNF for 1, 2 and 4 days [17]. More than 50% of the up-regulated genes in the study by Gurok were localized in chromosome regions that we found amplified in our present study. The amplified regions included 3 genes that showed the highest up-regulation of expression including 6330403K07 RIKEN cDNA, complement component 3 and complement component 4 cDNAs. Regarding the protein level we showed that amplification of the chromosome region harboring the *GFAP* gene was associated with high GFAP expression detectable by immune fluorescence as early as 12 hours after FCS differentiation induction. Likewise, we found high GFAP expression in TGF-ß differentiation induced SFME cells. In mesencephalon neurosphere cells we confirmed *GFAP* amplification and GFAP expression using simultaneous FISH and immunefluorescence analysis after 2-day differentiation induction. Considering the biological effects of the amplifications, one has to bear in mind that array-CGH is likely to miss chromosome regions that are amplified during differentiation. Since the array-CGH data stem from a mixed population of cells, this method indentified only amplifications that are present in at least 5% of the cells. In addition, array-CGH analysis does not show if a given amplified domain has the same extend in the cells that harbor this amplification. Thus, the *in vitro* array-CGH analysis can only be a first step towards a complete picture of gene amplifications in differentiating cells. We used fluorescence-*in-situ* hybridization on differentiation induced SFME cells and mesencephanlon derived primary neuronal progenitor cells to validate our CGH-analysis. Six chromosome regions randomly selected from the amplified chromosome regions were confirmed in our *in-situ* hybridization experiments. The FISH experiments showed several cases with a sprinkled highly dense pattern of fluorescence signals, which is probably due to amplification on double minutes or even smaller units like episomes. As for the amplification mechanism, the process underlying the amplification in normal differentiating cells is likely different from the amplification mechanism assumed for mammalian tumor cells. The breakage-fusion-bridge-cycle model that may explain gene amplifications in tumor cells is not able to explain the tremendous increase in gene copy numbers observed in differentiating cells within 12–48 hours. The observed increase points to a mechanism independent from mitosis and appears more related to the endocycling and amplification mechanism described in *Drosophila*. A recent study reports amplified and under-replicated regions caused by repression of replication initiation and fork progression in *Drosophila* [18].

Our *in vivo* results on gene amplifications in a specific mouse embryonic stage provide further evidence that developmental gene amplification is not restricted to amphibians and flies. The tissue section contains a heterogeneous mixture of cells at different time points of the differentiation process including stem cells, progenitor cells and completely differentiated cells. We showed that gene amplification is present in specific cells of the developing mouse brain at stage E11.5 of embryonic development. We assume that amplification occurs in cells in the process of differentiation. Notably, fluorescence *in situ* hybridizations was done on tissue sections with non-repetitive hybridization probes. Under these conditions signal detection is complicated by the thickness of the section and the possibility that nuclei are cut in two. This may cause possible loss of one of the signals of the two gene copies. Higher numbers of gene copies as result of an amplification process are, however, likely to be detected by strong hybridization signals even under these experimental conditions. Independent of these technical issues, it remains to be seen to what extend gene amplifications may be found beyond the analyzed stage, genes and cell types. Published array-CGH data of previous studies on haploid embryonic stem cells, murine iPS or tissues containing differentiating cells show a striking similar wavy CGH pattern that we found in our experiments. As addressed above, the wavy pattern appears to result neither from short protein digestion nor simply from replication timing. Throughout our studies, samples from murine or human neural stem cells were processed the same way and amplifications were detectable at various time points during differentiation. We are aware that our threshold setting is below the setting used in other studies, but mixed populations of cells with only small number of cells harboring amplifications require this setting to allow for amplification detection. In conclusion we need a rethinking on the genomic stability of stem cells. It is very unlikely that amplification as a powerful mean to up-regulate genes was lost during evolution from amphibians and flies towards mammals and only reinvented by tumor cells. Stem cells during differentiation might represent the link to explain the appearance of gene amplifications during tumor development.

## METHODS

### Cell culture and differentiation

SFME cells (CRL-9392™) were obtained from ATCC as cryopreserved culture and were cultivated in DMEM:F12 Medium supplemented with bovine insulin (0.01 mg/ml), human transferrin (0.01 mg/ml), chemically defined lipids (1%), sodium selenite (10 nM) and mouse EGF (50 ng/ml). Cells were seeded on fibronectin coated cultureware and allowed to grow for 18 h prior to differentiation induction with TGF-β. SFME cells were differentiation induced using above supplemented ATCC DMEM:F12 Medium containing TGF-β (10 ng/ml) for 24 h or DMEM:F12 supplemented with FCS for 12 h.

For fibronectin control experiments cells were grown an additional 4 d and 1 d on fibronectin before DNA isolation. Cells cultured in the absence of fibronectin formed spheres and served as non-differentiated controls.

Mouse Ventral Mesencephalon E14 neurospheres were obtained as P1 cryopreserved neurospheres from STEMCELL Technologies. These neurospheres contain neural stem and progenitor cells. Cells were cultured in complete NeuroCult^TM^ NSC proliferation medium supplemented with rhEGF (20 ng/ml). As undifferentiated controls those P1 neural stem and progenitor cells were cultivated on laminin coated glass slides for 2 d.

Differentiation was induced according to the manufacturer's instructions using NeuroCult^TM^ NSC differentiation medium without cytokines and with laminin or poly-D-lysine/laminin coated glass slides. Cells were differentiation induced for 2 d and 3 d. All experiments were done with neural stem and progenitor cells in P1 or P2. Cells differentiating for 3 d were cultured for 24 h in proliferation medium containing bFGF and EGF prior to differentiation induction. Culturing for 24 h in proliferation medium with bFGF and EGF is recommended by the manufacturers to stimulate progenitor cell proliferation.

### Array preparation, hybridization and detection

Genomic DNA was isolated as described previously [5] and mouse genomic DNA from Clontech was used as control DNA. NimbleGen 3×720K mouse whole genome array hybridization was performed with the certified full service of NimbleGen provided by ImaGenes Berlin, Germany. Array data were deposited in GEO (GSE35523).

### Fluorescence *in situ* hybridization

BAC clones were taken from the RP-11 (http://www.chori.org/bacpac/) libraries of the Welcome Trust Sanger Institute and available from ImaGenes GmbH, Germany.

BAC probes for *C1QL1* and *S1PR5* were directly labeled using the High Prime Labeling System (Roche Molecular Biochemicals, Germany). BAC-DNA (1 μg) was labeled with Cyanine-3-dCTP (Cy3) (pink fluorescence signals) according to the manufacturer's instructions. Additional BAC probes were either labeled with Alexa-488-dCTP (green fluorescence signals) or with Alexa-594-dCTP (pink fluorescence signals) using the FISHTag DNA labeling Kit according to the manufacturer's instructions. For multiple amplification analysis three BAC probes were labeled with Alexa-555-dCTP, with Alexa-488-dCTP and with Alexa-594-dCTP.

Differentially labeled probe DNAs (60 ng) were precipitated in the presence of mouse Cot-1 DNA. Samples were resuspended in hybridization mix (50% formamide, 2xSSPE, 10% dextrane sulphate and 4% SDS).

### Fluorescence *in situ* hybridization using SFME cells

SFME interphase nuclei were fixed using Carnoy's fixative (methanol ice acetic acid). Nuclei were dropped on clean glass slides. Slides were RNase treated (100 μg/ml RNaseA in 2 × SSC) for 30 minutes at 37°C and pepsin treated (0.005% in 0.01 M HCl at 37°C) for 10 minutes. Postfixation was performed using 1% formaldehyde/1x PBS for 10 minutes at room temperature.

Labeled BAC probes were applied to the slides and denatured for 2 min at 80°C. Hybridization was carried out in a humid chamber at 37°C for 16 h. Post hybridization washes were performed in 50% formamide/2 × SSPE (4 × 5 minutes; 45°C) followed by 0.1 × SSPE (3 × 5 minutes) at 60°C. Nuclei were counterstained with DAPI (4′,6′-Diamidino-2-phenylindole) (1 μg/ml in PBS) for 4 minutes and mounted with VectaShield mounting medium (Vector Laboratories, Orton Southgate, England) for microscopic analysis.

### Fluorescence *in situ* hybridization on neural stem and progenitor cells

Differentiating neural stem and progenitor cells (primary mesencephalon E14 neurosphere cells) on coated glass slides were fixed in ice-cold methanol for 20 minutes. Slides were washed in PBS for 5 minutes and treated with 0.02% Tween-20 for 5 minutes. Slides were treated with RNaseA for 45 minutes, digested with pepsin and fixed with formaldehyde/1xPBS for 10 minutes. Hybridization and post hybridization washes were as described above.

### Fluorescence *in situ* hybridization on mouse embryo cryosections

Transverse cryosections (10 μm) from mouse brain at embryonic stage E11.5 were treated with Carnoy's fixative for 15 min at 4°C, pepsin digested and fixed 4% paraformaldehyde in diethylpyrocarbonate-treated PBS for 10 minutes.

Probes were labeled as described above, applied to the section and denatured for 5 min at 80°C. Hybridization was for 2–3 days at 37°C. Posthybridization washes and DAPI staining were as described above.

### Fluorescence *in situ* hybridization with simultaneous immune fluorescence

Differentiating neural stem and progenitor cells (primary mesencephalon E14 neurosphere cells) on coated glass slides were fixed in ice-cold methanol for 20 minutes. Slides were washed in PBS for 5 minutes and treated with 0.2% Tween-20 for 5 minutes. Postfixation was done by 1% formaldehyde/1x PBS for 10 minutes at room temperature. Slides were blocked with goat serum and incubated for 1 h with chicken antibody polyclonal to GFAP (ab4674, Abcam) and detected using an Alexa-488 coupled secondary antibody. Finally, slides were dehydrated by an ascending ethanol series (70%/80%/96%) and air-dried. Hybridization and post hybridization washes were as described above.

### Immune fluorescence

SFME cells were cultivated on fibronectin-coated glass coverslips. Subsequently, cells were differentiation induced as described above. Both induced cells and untreated controls were methanol fixed and treated with 0.2% Tween-20 for 2 minutes. Coverslips were blocked with goat serum and incubated for 1 h with chicken antibody polyclonal to GFAP (ab4674, Abcam) and rabbit antibody polyclonal to nestin (ab27952, Abcam). Detection was done with an Alexa-488 coupled secondary antibody against chicken and Alex-594 coupled secondary antibody against rabbit.

Differentiating neural stem and progenitor cells (primary mesencephalon E14 neurosphere cells) grown on coated glass slides were fixed in ice-cold methanol for 20 minutes. Slides were washed in PBS for 5 minutes and treated with 0.2% Tween-20 for 2 minutes. Subsequently, slides were blocked with goat serum and incubated for 1 h with chicken antibody polyclonal to GFAP (ab4674, Abcam) and rabbit antibody polyclonal to nestin. Detection was done with an Alexa-488 coupled secondary antibody against chicken and Alex-594 coupled secondary antibody against rabbit.

### Microscope imaging and analysis

Fluorescence images were captured with an Olympus AX70 microscope using ISIS software from Metasystems. For an overview of DAPI stained cryosections, images were taken at an Olympus BX61 microscope. For histological staining, cryosections of 10 μm thickness were fixed in acetone at –20°C for 10 min and stained in luxol fast blue solution at 56°C overnight, followed by cresyl violet staining. Histological images were taken at an Olympus BX51 TF microscope.

## SUPPLEMENTARY FIGURE


