# Supplementary material for: Gene amplification during differentiation of mammalian neural stem cells in vitro and in vivo
Source: Oncotarget. 2015 Feb 19;6(9):7023–39. doi: 10.18632/oncotarget.3248 (PMC4466667; doi:10.18632/oncotarget.3248)
Supplement: Supplementary file 1 [file oncotarget-06-7023-s001.pdf]

SUPPLEMENTARY FIGURE

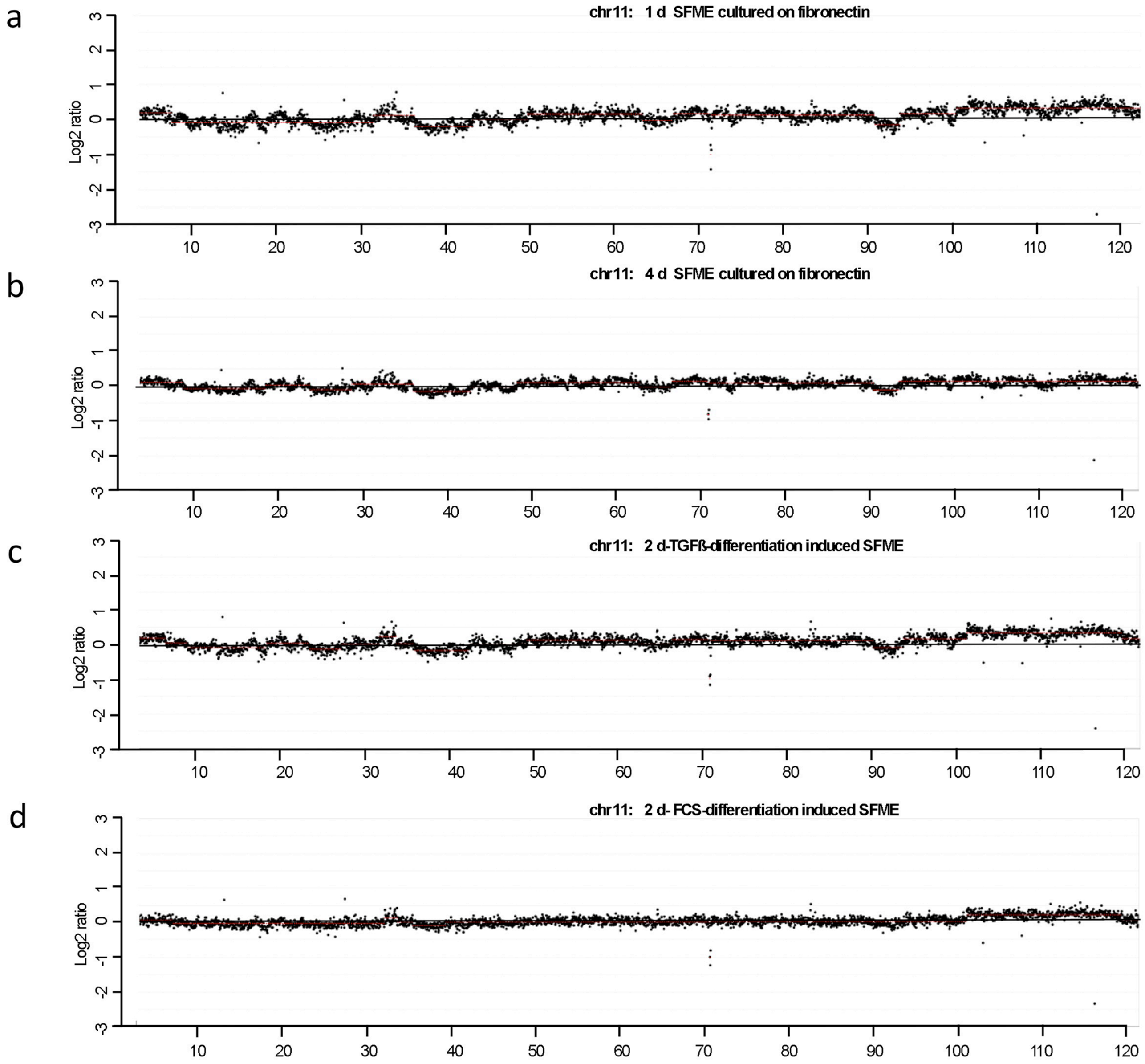

Supplementary Figure S1: Additional Chromosome 11 plots. Representative chromosome 11 plots at 40kb resolution using a log_2 scale from undifferentiated SFME cells cultivated on fibronectin for 1d **(a)** or 4d **(b)**, 2d-TGF-ß-differentiation induced SFME cells **(c)** and 2d-FCS-differentiation induced SFME cells **(d)**.
